# Supplementary material for: Content-rich biological network constructed by mining PubMed abstracts
Source: BMC Bioinformatics. 2004 Oct 8;5:147. doi: 10.1186/1471-2105-5-147 (PMC528731; doi:10.1186/1471-2105-5-147)
Supplement: Additional File 2 — The original results of the above study (non-essential files are deleted to keep the file size under the limit set by BMC bioinformatics). [file 1471-2105-5-147-S2.bz2 › chilibotAdditionalFile2/dip50/142ID7739537E502/html/RBL1_TFDP1.html]

 


 **RBL1** and **TFDP1** 
  
Found 64 abstracts in PubMed, retrieved 50.  
 

 What does Google say? 
 PDF only 
| .edu only 

---

**Interactive relationship** (e.g. stimulation, inhibition, etc)

**Inhibitory relationship**- Reinduction in quiescent cells of DNA synthesis by E2F1 DP1  [ **TFDP1** ]  overexpression is abrogated by coexpression of pRb  [ **RBL1** ]  and is hampered by MyoD overexpression.  Ref: 9537223 Cancer Res, 1998
- We report here that transfection of cells with the known components of this complex, p107  [ **RBL1** ] , E2F 4 and DP 1  [ **TFDP1** ] , did not repress the B myb promoter in cycling NIH3T3 cells, although p107  [ **RBL1** ]  inhibited transcription transactivation by E2F 4 DP 1  [ **TFDP1** ] .  Ref: 8806697 Oncogene, 1996
- We found that anti IgM treatment induces the appearance of an inhibitory DNA binding complex containing the pRB  [ **RBL1** ]  related pocket protein p130 together with E2F and a concomitant decrease in free E2F, consisting of E2F1 and its partner DP1  [ **TFDP1** ] .  Ref: 9545352 J Biol Chem, 1998
- DRTF1  [ **TFDP1** ]  E2F is believed to coordinate and integrate the transcription of cell cycle regulating genes, for example, those involved in DNA synthesis, with the activity of regulatory proteins, such as the retinoblastoma tumor suppressor gene product pRb  [ **RBL1** ] , which modulate its transcriptional activity.  Ref: 8816502 Mol Cell Biol, 1996
**Neutral relationship**- When DP1  [ **TFDP1** ]  was connected to cyclin A Cdk2 through E2F4 and p107  [ **RBL1** ] , its phosphorylation was very inefficient, although its association with cyclin A Cdk2 was stable.  Ref: 10329431 Biochem Biophys Res Commun, 1999
- In contrast, the p107  [ **RBL1** ]  p130 associated E2Fs require the coexpression of the heterodimeric partner DP 1  [ **TFDP1** ]  to promote S phase entry and accelerate G1 progression.  Ref: 8622649 Mol Cell Biol, 1996
- Our results establish DRTF1  [ **TFDP1** ]  E2F as a common cellular target in growth control mediated through the activities of pRb  [ **RBL1** ]  and p53 and suggest an alternative mechanism through which p53 may regulate cellular proliferation.  Ref: 8816502 Mol Cell Biol, 1996
- During late G1 and S phase this complex is replaced by at least three different E2F complexes, one of which is an E2F complex containing p107  [ **RBL1** ]  or pRB  [ **RBL1** ]  as well as two free E2F complexes consisting of E2F4 DP1  [ **TFDP1** ]  and E2F1 3 DP1  [ **TFDP1** ] .  Ref: 10540350 Eur J Immunol, 1999
- DP1  [ **TFDP1** ]  phosphorylation in multimeric complexes weaker interaction with cyclin A through the E2F1 cyclin A binding domain leads to more efficient phosphorylation than stronger interaction through the p107  [ **RBL1** ]  cyclin A binding domain.  Ref: 10329431 Biochem Biophys Res Commun, 1999

**Non-interactive relationship** (e.g. studied together, co-existance, homology, etc.)

- Antibodies to DP 1  [ **TFDP1** ] , E2F1 to 5, p107  [ **RBL1** ] , or pRb  [ **RBL1** ]  failed to either supershift or block E2Fmyb sp complex formation.  Ref: 10567569 Mol Cell Biol, 1999
- In serum stimulated S phase cells, the composition of the complex switched to E2F1 4, DP1  [ **TFDP1** ]  and p107  [ **RBL1** ] , together with cyclin A and cyclin dependent kinase 2.  Ref: 12139485 Biochem J, 2002
- E2F1, the first cloned member of this family, is regulated during the cell cycle at the mRNA level by changes in transcription of the E2F1 gene and at the protein level by complex formation with proteins such as the retinoblastoma gene product pRB  [ **RBL1** ] , cyclin A and DP1  [ **TFDP1** ] .  Ref: 8964493 Gene, 1996
- As cells enter G1, E2F 4 is dephosphorylated to several hypophosphorylated forms and three new DNA binding complexes appear, including one containing E2F 4, DP 1  [ **TFDP1** ] , and p107  [ **RBL1** ] .  Ref: 9207453 Blood, 1997
- Cotransfection of expression vectors encoding p107  [ **RBL1** ] , p130, or DP 2, but not DP 1  [ **TFDP1** ] , resulted in the nuclear localization of E2F 4 and 5.  Ref: 9144196 Proc Natl Acad Sci U S A, 1997
- Because Cdk4 6 phosphorylates retinoblastoma protein pRb  [ **RBL1** ]  family members that then modulate the transcriptional activity of E2F DP1  [ **TFDP1** ]  complexes, we examined the involvement of these components in DNA damage evoked neuronal death.  Ref: 10777774 J Neurosci, 2000
- Expression of pRB  [ **RBL1** ] , cyclin cyclin dependent kinases and E2F1 DP 1  [ **TFDP1** ]  in human tumor lines in cell culture and in xenograft tissues and response to cell cycle agents.  Ref: 11052627 Cancer Chemother Pharmacol, 2000
- v Abl tyrosine kinase dependent changes in proteins binding the c myc E2F site were also demonstrated, including induction of a complex containing DP1  [ **TFDP1** ] , p107  [ **RBL1** ] , cyclin A, and cdk2.  Ref: 8524218 Mol Cell Biol, 1995
- Moreover, introduction of constitutively active pRb  [ **RBL1** ]  and transcriptionally inert mutant E2F1 DP1  [ **TFDP1** ]  efficiently protected cardiomyocytes from apoptosis.  Ref: 12411392 Circ Res, 2002
- Together with the ability of cyclin E to overcome a G1 block induced by expression of dominant negative mutant DP 1  [ **TFDP1** ] , a heterodimeric partner of E2Fs, these results provide evidence for a cyclin E controlled S phase promoting event in somatic cells downstream of or parallel to phosphorylation of pRb  [ **RBL1** ]  and independent of E2F activation.  Ref: 9192874 Genes Dev, 1997
- Western blot analyses of whole cell extracts revealed that amounts of E2F4, E2F1, DP1  [ **TFDP1** ] , and p107  [ **RBL1** ]  remained unchanged after infection of C33 cells.  Ref: 10366563 Virology, 1999
- Furthermore, E2F species were sequestered in complexes with p107  [ **RBL1** ] , p130, DP 1  [ **TFDP1** ] , and cyclins A and E.  Ref: 12234612 Biochem Pharmacol, 2002
- A complex containing Smad3, the transcription factors E2F4 5 and DP1  [ **TFDP1** ] , and the corepressor p107  [ **RBL1** ]  preexists in the cytoplasm.  Ref: 12150994 Cell, 2002
- We have shown that p202, an IFN inducible 52kDa primarily nuclear phosphoprotein whose expression in transfected cells inhibits cell proliferation, interacts with the retinoblastoma tumor suppressor protein pRb  [ **RBL1** ]  and the transcription factor E2F E2F 1 DP 1  [ **TFDP1** ]  in vitro and in vivo.  Ref: 9233764 Oncogene, 1997
- Expression of pRb  [ **RBL1** ] , cyclin D1, cdk4, cyclin E, cdk2, E2F1 and DP 1  [ **TFDP1** ]  was determined in MCF 7 and MDA MB 468 breast carcinoma cells, H460 and Calu 6 non small cell lung carcinoma cells, H82 and SW2 small cell lung carcinoma cells, HCT116 and HT29 colon carcinoma cells and LNCaP and DU 145 prostate carcinoma cells.  Ref: 11052627 Cancer Chemother Pharmacol, 2000
- Release of E2F1 DP1  [ **TFDP1** ]  heterodimers from repression mediated by the retinoblastoma tumor suppressor pRB  [ **RBL1** ]  triggers cell cycle entry into S phase, suggesting that E2F1 and DP1  [ **TFDP1** ]  proteins must act in unison, either to facilitate or to suppress cell cycle progression.  Ref: 12588846 Development, 2003
- Overexpression of c myc, Cyclins D1, E, and A, and E2F1 genes, at messenger RNA mRNA and protein levels, rise in Cyclin D1 CDK4, Cyclin E CDK2, and E2F1 DP1  [ **TFDP1** ]  complexes, and pRb  [ **RBL1** ]  hyperphosphorylation occurred in nodules and HCCs of F344 rats.  Ref: 12029619 Hepatology, 2002
- Severe deregulation of G1 S transition was also indicated by the dramatic up regulation, particularly in the HCCs, of pRb  [ **RBL1** ]  free E2F1 DP1  [ **TFDP1** ]  and E2F2 DP1  [ **TFDP1** ]  transcription factor heterodimers, as assessed by immunoprecipitation and immunohistochemistry.  Ref: 9426068 Cancer Res, 1998
- Intriguingly, the major E2F complex that we detected in quiescent human B lymphocytes is consisted of pRB  [ **RBL1** ] , E2F4, and DP1  [ **TFDP1** ] .  Ref: 10207023 J Biol Chem, 1999
